# On the interpretability and computational reliability of frequency-domain Granger causality

**DOI:** 10.12688/f1000research.12694.1

**Published:** 2017-09-20

**Authors:** Luca Faes, Sebastiano Stramaglia, Daniele Marinazzo

**Affiliations:** 1 BIOtech, Department of Industrial Engineering, University of Trento, Trento, Italy; 2 Istituto Nazionale di Fisica Nucleare, Sezione di Bari, Bari, Italy; 3 Dipartimento di Fisica, Università degli Studi Aldo Moro, Bari, Italy; 4 Department of Data Analysis, Ghent University, Ghent, Belgium

**Keywords:** Granger-Geweke causality, frequency-domain connectivity, time series analysis, directed coherence, vector autoregressive models, spectral decomposition, brain connectivity, physiological oscillations

## Abstract

This Correspondence article is a comment which directly relates to the paper “A study of problems encountered in Granger causality analysis from a neuroscience perspective” ( Stokes and Purdon, 2017). We agree that interpretation issues of Granger causality (GC) in neuroscience exist, partially due to the historically unfortunate use of the name “causality”, as described in previous literature. On the other hand, we think that Stokes and Purdon use a formulation of GC which is outdated (albeit still used) and do not fully account for the potential of the different frequency-domain versions of GC; in doing so, their paper dismisses GC measures based on a suboptimal use of them. Furthermore, since data from simulated systems are used, the pitfalls that are found with the used formulation are intended to be general, and not limited to neuroscience. It would be a pity if this paper, even if written in good faith, became a wildcard against all possible applications of GC, regardless of the large body of work recently published which aims to address faults in methodology and interpretation. In order to provide a balanced view, we replicate the simulations of Stokes and Purdon, using an updated GC implementation and exploiting the combination of spectral and causal information, showing that in this way the pitfalls are mitigated or directly solved.

## Correspondence

Granger causality (GC) ^1^ is an extremely popular statistical tool used to analyze directed interactions from multivariate time series measured from coupled dynamical systems. A particularly appealing aspect of the notion of GC is that it can be formulated in the frequency domain, and is thus eligible for the analysis of signals that are rich of oscillatory content such as those commonly encountered in neuroscience and physiology ^2, 3^. The spectral formulation of GC is obtained by elaborating in the frequency domain the parameters of the linear vector autoregressive (VAR) model that fit the observed multivariate time series. A main approach to do this was developed by Geweke ^4, 5^, yielding bivariate and conditional frequency domain measures of the so-called Granger-Geweke causality (GGC). An alternative framework stems from the works of Kaminski *et al* ^6^. and Baccalà *et al* ^7, 8^., who derived measures like the directed coherence (DC) and the partial DC (PDC), quantifying the total and direct directed influence of one time series over another in a fully multivariate setting (see references 9– 11 for comprehensive treatments).

In their recent work ^12^, Stokes and Purdon performed a critical evaluation of frequency-domain GC computed within the Geweke framework, evidencing in two simulation studies some computational and interpretational problems associated with the GGC measures. Specifically, they showed that – even when the systems generating the observed data belong to the finite-order VAR model class – spectral GGC cannot be reliably estimated and cannot recover the functional oscillatory structure underlying the data. These observations led the authors to conclude that the notion of causality quantified by GGC, and by other Granger causality measures in general, often yield counterintuitive and misleading results, thus being incompatible with the objectives of many neuroscience studies.

We definitely agree that GC and lag-based data-driven methods in general cannot provide measures of “causality” as intended in other applications (see references 9 and 13 for a thoughtful distinction between data-driven and model-based approaches). We also share the view that the assumptions of linearity and stationarity, as well as the presence of unobserved variables, noise or inappropriate sampling may pose theoretical and practical problems which can severely impair both the formulation and the computation of spectral GC measures – this has been stated by Stokes and Purdon ^12^ and in previous studies ^2, 3, 14^. On the other hand we think that, based on the way simulated data have been analyzed and interpreted by Stokes and Purdon ^12^, frequency domain GC methods have been dismissed based on a suboptimal (even though frequently applied) formulation of GGC, and based on the lack of direct consideration of the DC/PDC framework.

In this contribution, we repeat the simulations of Stokes and Purdon ^12^, and suggest that the negative conclusions based on the results of such simulations are overstated. We show that spectral GGC estimates can be obtained with a high computational reliability if proper estimation approaches are employed, and the interpretation of frequency domain causality measures can be meaningfully performed if spectral and causal information are properly combined. The codes for running our analyses are based on existing Matlab ^©^ toolboxes ^11, 15, 16^ and are provided as supplementary data alongside this article.

**Figure 1.**
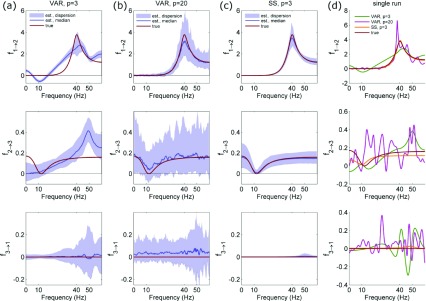
Comparison of conditional frequency-domain Granger-Geweke causality (GGC) profiles computed for the three-node system of Stokes and Purdon ^12^ (Example 1, where nodes 1, 2, and 3 resonate respectively at 40 Hz, 10 Hz, and 50 Hz, and where unidirectional causality is imposed from node 1 to node 2, and from node 2 to node 3). GGC is computed along the two coupled directions (f _1→2_, f _2→3_) and along a direction with no coupling (f _3→1_). Columns report the distribution of GGC estimates (median and 5 ^th^–95 ^th^ percentiles) computed using classical vector autoregressive (VAR) estimation of full and reduced models performed with the true model order p=3 ( **a**) and with an increased order p=20 ( **b**), and using state space (SS) estimation ( **c**), as well as estimates obtained for a single simulation run ( **d**); in each plot, the true causality values computed from the original model parameters are reported in red. Results evidence the lower bias and variability of spectral GGC computed using the SS method compared to the classical VAR approach.

The first simulation of Stokes and Purdon ^12^ shows that, due to the modeling choices required to compute spectral GGC, this measure cannot be reliably estimated even for simple systems. By generating 100 realizations of this simulation with the same parameters and data length we confirm that, by applying the standard method of fitting separate full and reduced VAR models, spectral GGC estimates display a strong bias ( Figure 1A) or a very large variability ( Figure 1B), depending on the choice of the model order. As explained by Stokes and Purdon ^12^, this tradeoff between bias and variance arises from the incorrect representation of the reduced model as a VAR process of finite order. Exactly for this reason however, the problem can be overcome employing the state-space (SS) approach ^16^, which allows to compute GGC in closed form from the SS parameters of any observed VAR process. Here we show that this approach yields highly accurate spectral estimates of GGC, which closely follow the expected profiles over the coupled directions and have negligible magnitude over the uncoupled direction ( Figure 1C); the higher reliability of the SS estimator compared with the standard VAR-based method is evident also looking at single process realizations ( Figure 1D).

**Figure 2.**
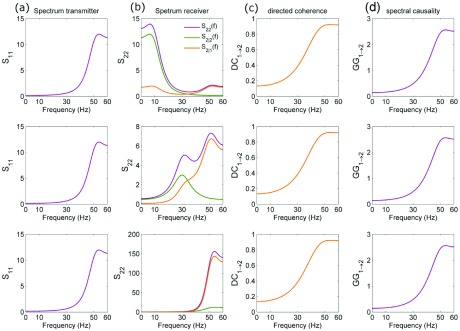
Theoretical profiles of spectral and causality measures computed for the two-node system of Stokes and Purdon ^12^ (Example 2, where unidirectional causality is imposed from node 1 to node 2). The system is studied setting a resonance frequency of 50 Hz for the transmitter and of 10 Hz (top row panels), 30 Hz (mid row panels) and 50 Hz (bottom row panels) for the receiver. The fact that the power spectral density (PSD) of the transmitter (S _11_(f), **a**) is the same for the three cases induces, together with the unaltered coefficients determining the causal effects, the same profile for the directed coherence DC _1→2_(f) ( **c**) and the spectral GGC measure GG _1→2_(f) ( **d**). However, the different causal contribution of the transmitter on the receiver is revealed by the partial spectrum S _2|1_(f)=S _22_(f)·DC _1→2_(f) (orange line in ( **b**)), which quantifies the portion of the overall PSD of the receiver (S _22_(f), purple line in ( **b**)) that is causally explained by the transmitter; the non-explained part (S _2|2_(f), green line in ( **b**)) reflects the autonomous dynamics of the receiver.

The second simulation of Stokes and Purdon ^12^ shows that, due to the independence of GGC measures from the intrinsic dynamics of the “receiver” process, the spectral GGC profiles linking this process to its putatively causal “transmitter” process are often misleading, because different systems can have identical causality functions but different receiver dynamics. In Figure 2 we confirm this result both in terms of GGC and using the DC, a spectral causality measure taken from the VAR framework ^7^ that for bivariate processes like the one simulated here is analytically related to the spectral GGC ^15^. However, this invariance property is in our view absolutely reasonable, because the DC has a clear-cut interpretation as the relative amount of spectral power that, at each frequency, arrives to the receiver starting from the transmitter ^11^. Nevertheless, the DC is also useful to fully recover the functional oscillatory structure of the observed processes, because it shapes the receiver spectrum to reveal the portion of its spectral power that is “causally” due to the transmitter; this is depicted in the spectral decomposition of Figure 2.

In conclusion, while thanking Stokes and Purdon ^12^ for pointing out some weaknesses of GGC measures, we think that proper formulations can provide meaningful results of directed dynamical influence, whose interpretation still is bound to the knowledge and good faith of those who write and read related scientific literature.

Codes to compute frequency-domain Granger causality in linear stochastic processes The package contains Matlab scripts and functions that allow to reproduce the simulations of Stokes and Purdon, and to compare for these simulations: (i) the standard vector autoregressive estimator and the updated state space estimator of the spectral Granger-Geweke causality measure; (ii) the spectral Granger-Geweke causality measure with the directed coherence measure. Click here for additional data file. Copyright: © 2017 Faes L et al. 2017 Data associated with the article are available under the terms of the Creative Commons Zero "No rights reserved" data waiver (CC0 1.0 Public domain dedication).

## Data availability

The data referenced by this article are under copyright with the following copyright statement: Copyright: © 2017 Faes L et al.

Data associated with the article are available under the terms of the Creative Commons Zero "No rights reserved" data waiver (CC0 1.0 Public domain dedication). 

**Dataset 1: Codes to compute frequency-domain Granger causality in linear stochastic processes.** The package contains Matlab scripts and functions that allow to reproduce the simulations of Stokes and Purdon, and to compare for these simulations: (i) the standard vector autoregressive estimator and the updated state space estimator of the spectral Granger-Geweke causality measure; (ii) the spectral Granger-Geweke causality measure with the directed coherence measure.

DOI, 10.5256/f1000research.12694.d178159 ^17^.
