# Supplementary material for: On the interpretability and computational reliability of frequency-domain Granger causality
Source: F1000Res. 2017 Sep 20;6:1710. [Version 1] doi: 10.12688/f1000research.12694.1 (PMC5676195; doi:10.12688/f1000research.12694.1)
Supplement: Peer review history

# Peer review: Referee response for version 1

**DOI:** 10.5256/f1000research.13747.r26209

**Published:** 2017-11-06

**Authors:** Karin Schiecke

**Affiliations:** 1 Bernstein Group for Computational Neuroscience Jena, Institute of Medical Statistics, Computer Sciences and Documentation (IMSID), Jena University Hospital, Friedrich Schiller University Jena, Jena, Germany

The correspondence is a comment directly related to “A study of problems encountered in Granger causality analysis from a neuroscience perspective ^1^. I completely agree with the authors that there are interpretation issues of Granger causality (and by the way also a lot of wrong use/wrong adaptation in the field) and therefore the whole concept has to be applied with care. But – like the authors clearly stated in their comment – Stokes and Purdon uses 1) a version of frequency-domain Granger causality which is a least sub-optimal and 2) the results they were able to show by means of simulated data should be a general problem not only occurring in the case of neuroscience data. Therefore, I explicitly support the comment of Faes, Stramaglia and Marinazzo.

 The comment is well written. Basic information concerning the concept of Granger causality and in particular the frequency-domain approach are given (including the coverage of related literature). The main idea behind the paper of Stokes and Purdon and the reason why the authors felt the need to comment on this paper are clearly stated. Then, the simulations of Stokes and Purdon are repeated using an updated Granger causality implementation and a combination of spectral and causal information. Technical details are given, results are correctly presented and well described, and conclusions are drawn.

 I do not have any major or minor concerns.

I have read this submission. I believe that I have an appropriate level of expertise to confirm that it is of an acceptable scientific standard.

## References

[1] StokesPAPurdonPL: A study of problems encountered in Granger causality analysis from a neuroscience perspective. *Proc Natl Acad Sci U S A*.2017;114(34) : 10.1073/pnas.1704663114 E7063-E7072 10.1073/pnas.1704663114 28778996PMC5576801

# Peer review: Referee response for version 1

**DOI:** 10.5256/f1000research.13747.r26204

**Published:** 2017-11-02

**Authors:** Luiz Antonio Baccala

**Affiliations:** 1 Department of Telecommunications and Control Engineering, Polytechnic School, University of São Paulo, São Paulo, Brazil

I am glad that Faes et al. have not fallen prey to these considerations and have mounted a fair and elegant, albeit brief, critical appraisal of the PNAS Stokes and Purdon (S/P) paper. Fael et al. have restricted themselves to nailing just three obvious crucial facts that may go unnoticed by the casual reader for whom the authoritativeness of a vehicle like PNAS may stand as a certificate of validity.

 The first point is that the S/P paper fails to  mention how their VAR models were computed. My own AsympPDC package (The AsympPDC Package 3.0 is directly downloadable from http://www.lcs.poli.usp.br/~baccala/pdc/asymp_package_v3.zip. A preliminary version is also available through [7]. Visit http://www.lcs.poli.usp.br/~baccala/pdc/ for future version updates.) provides five different methods: the simplest naive and most popular  approach, the least squares method, is the worst performer thanks to error propagation alone. It is important to stress that accurate VAR model estimation is crucial to whichever approach to GC is chosen. Faes et. al correctly  supply an alternative method of estimation where no theoretically meaningless ‘negative’ GGC value is observed. The Faes et al. paper has the added obvious merit of including the actual value of GGC computed from the actual true model absentmindedly lacking in the  original S/P paper.

 The second point raised almost in passing by Faes et al. is that the S/P paper may possibly induce its readers to completely disregard frequency domain causality descriptions while S/P mostly glosses over the alternative DC/PDC framework. This is a huge oversight since currently only DC/PDC estimators have statistical theoretically rigorous computable confidence intervals and objective null hypothesis threshold tests ^1^^-^^2^ which may be applied using the freely downloadable AsympPDC package. This fact alone sets the DC/PDC methodologically apart.  In today’s internet era of information no more than a google away, this omission is unforgivable.

 The third point concerns the two time series case of Example 2. Indeed here is a point that Faes et al correctly argue that S/P fail to grasp. GC was conceived by Granger ^3^ to decompose pairwise relationships into exposing factors that aid predictability. This was later shown to be equivalent to  detecting and characterizing the presence of feedback by Sims ^4^. In this case, GGC, DC and PDC address the so called connection ‘detection’ problem by focusing on the connection.  If some given influence is present and if an influenced subsystem is affected, how it responds resonantly or otherwise is its own business.

 Do the valid Faes et al. criticisms imply the S/P paper is worthless?  Despite its many additional shortcomings, the S/P paper has the important merit of stripping bare some of  the field’s reigning confusion, enough to call for added discussion. This only stresses the relevance of the present criticisms and the urgent need for clarification.

 Finally I would like to put forward some thoughts that may explain the present state of conceptual disarray regarding causality. The first issue is ignorance about time series estimation —  of the ‘a little knowledge is dangerous thing' kind.  One cannot expect correct and reasonable results by just downloading some package, pressing some buttons without knowing the precise limitations of each available tool - time series analysis still has some elements of an art. The second problem has to do with the notions of Causality - it is tempting to ask the methodology to provide a glimpse on actual mechanisms. Sometimes, on physical grounds, this desire may be fulfilled, but true (mechanical) causality determination requires observer intervention ^5^^-^^6^;  what GC does is to allow the exclusion of some tentative mechanisms. Thirdly, even when it reflects actual mechanisms, but more than two time series are simultaneously examined, different descriptions, as is the case for the complementarity between DC and PDC,  they reflect different system properties so that one must break the GC concept into more general ideas: Granger Connectivity and Granger Influentiability ^5^^-^^6^. Last but not least, popular descriptions of large scale connectivity sometimes qualified as ‘effective’ versus ‘functional’ have further added to the present state of confusion due to their shear inconsistent application throughout the literature (se  a discussion in ^5^^-^^7^.

 I think  we should thank Faes et al. for pointing out some of these problems. Perhaps this is a good opportunity to start clearing up these issues once and for all.

I have read this submission. I believe that I have an appropriate level of expertise to confirm that it is of an acceptable scientific standard.

## References

[1] BaccaláLAde BritoCSTakahashiDYSameshimaK: Unified asymptotic theory for all partial directed coherence forms. *Philos Trans A Math Phys Eng Sci*.2013;371(1997) : 10.1098/rsta.2012.0158 20120158 10.1098/rsta.2012.0158 23858488

[2] BaccalaLATakahashiDYSameshimaK: Directed Transfer Function: Unified Asymptotic Theory and Some of Its Implications. *IEEE Trans Biomed Eng*.2016;63(12) : 10.1109/TBME.2016.2550199 2450-2460 10.1109/TBME.2016.2550199 27076053

[3] GrangerC: Investigating Causal Relations by Econometric Models and Cross-spectral Methods. *Econometrica*.1969;37(3) : 10.2307/1912791 10.2307/1912791

[4] SimsC: Money, income, and causality. *The American Economic Review*.1972;540-552

[5] BaccaláLSameshimaK: Causality and Influentiability: The Need for Distinct Neural Connectivity Concepts.2014;8609: 10.1007/978-3-319-09891-3_39 424-435 10.1007/978-3-319-09891-3_39

[6] SameshimaKBaccalaLA: Methods in Brain Connectivity Inference through Multivariate Time Series Analysis. *CRC Press*.2014;

[7] BaccaláL. ASameshimaK: A neural link centered approach to brain connectivity. *Cognitive Science: Recent Advances and Recurring Problems, Vernon Press*.2017;101-112
